# Corneal Nerve Fiber Structure, Its Role in Corneal Function, and Its Changes in Corneal Diseases

**DOI:** 10.1155/2017/3242649

**Published:** 2017-11-07

**Authors:** Hiroshi Eguchi, Akio Hiura, Hiroshi Nakagawa, Shunji Kusaka, Yoshikazu Shimomura

**Affiliations:** ^1^Department of Ophthalmology, Kindai University Sakai Hospital, Osaka, Japan; ^2^Department of Oral Histology, School of Dentistry, Tokushima University, Tokushima, Japan; ^3^Department of Pediatric Dentistry, Tokushima University Hospital, Tokushima University, Tokushima, Japan; ^4^Department of Ophthalmology, Kindai University, Osaka, Japan

## Abstract

Recently, in vivo confocal microscopy is used to examine the human corneal nerve fibers morphology. Corneal nerve fiber architecture and its role are studied in healthy and pathological conditions. Corneal nerves of rats were studied by nonspecific acetylcholinesterase (NsAchE) staining. NsAchE-positive subepithelial (stromal) nerve fiber has been found to be insensitive to capsaicin. Besides, NsAchE-negative but capsaicin-sensitive subbasal nerve (leash) fibers formed thick mesh-like structure showing close interconnections and exhibit both isolectin B4- and transient receptor potential vanilloid channel 1- (TRPV1-) positive. TRPV1, TRPV3, TRPA (ankyrin) 1, and TRPM (melastatin) 8 are expressed in corneal nerve fibers. Besides the corneal nerve fibers, the expressions of TRPV (1, 3, and 4), TRPC (canonical) 4, and TRPM8 are demonstrated in the corneal epithelial cell membrane. The realization of the importance of TRP channels acting as polymodal sensors of environmental stresses has identified potential drug targets for corneal disease. The pathophysiological conditions of corneal diseases are associated with disruption of normal tissue innervation, especially capsaicin-sensitive small sensory nerve fibers. The relationships between subbasal corneal nerve fiber morphology and neurotrophic keratopathy in corneal diseases are well studied. The recommended treatment for neurotrophic keratopathy is administration of preservative free eye drops.

## 1. Introduction

The structure and distribution pattern of corneal nerves have been extensively studied by histochemical and in vivo confocal microscopic (IVCM) methods to date. Recently, the human corneal nerve fiber structure is comprehensively demonstrated on the anterior-cornea of whole mount preparation (WMP) from 16 donors aged 19–78 years using *β*-tubulin as a primary antibody [1]. Main thick stromal nerve bundles enter the cornea at the corneoscleral limbus and make midstromal plexus and dense subepithelial plexus (SEP), by repeatedly branching. Straight and curvilinear nerve fibers of the SEP penetrate Bowman's membrane and innervate corneal epithelium [1]. This distribution pattern of the corneal nerves seems to be a common feature as described below. However, a correlation between neuropeptides, isolectin B4 (IB4: a plant lectin specifically binds to small sensory neurons), or transient receptor potential (TRP) channels and a classic neurotransmitter acetylcholine is poorly understood. One of the aims of this review is trying to focus on this issue. Further, differences in the structures of corneal nerves between animals and human are referred to in the recent reports.

It is well known that the cornea is densely innervated. For example, the density of nerve fibers in the rabbit corneal epithelium was estimated as 300–600 times and 20–40 times compared to that of skin and tooth pulp, respectively [2]. Corneal nerves respond to many sensations such as pain, temperature, or touch and functions in corneal reflex (blink) and tear production [3]. Moreover, corneal nerves contain many neurotrophic factors such as substance P (SP), calcitonin gene-related peptide (CGRP), epidermal growth factor (EGF), nerve growth factor (NGF), brain-derived neurotrophic factor (BDNF), and neurotrophin- (NT-) 3 [3]. These substances are directly released from unmyelinated C-fibers according to the inflammatory conditions and have functions to sustain the normal cornea and corneal wound healing (neurotrophic function) [3]. Majority of innervation to cornea is considered to be nociceptive in nature [4]. Approximately 7000 nociceptors/mm^2^ are estimated to be present in the human corneal epithelium [5].

Corneal nerve dysfunction due to mechanical or chemical trauma, inflammation, refractive surgery, and infections frequently induces corneal diseases such as opacities suffering from impaired vision. Therefore, precise understanding of the corneal nerves in health and disease has a high clinical impact for the patients with various corneal abnormalities. On this subject, an understanding of the corneal nerve fiber structures of animals is momentous for human therapeutic criteria (regeneration of corneal nerves and epithelial cells). Moreover, the cellular and molecular changes within primary sensory neurons underlying nociception, wound healing, scarring, dry eye, and immune status of the cornea are raised with regard to the TRP channels.

## 2. Structure and Distribution of Corneal Nerves

Corneal nerves have been visualized by some histochemical staining methods with gold chloride (AuCl), cholinesterase enzymes, and immune-reaction of antigen (neuropeptides) to their antibodies. Although numerous neurotransmitters and neuropeptides are found in the cornea and tear fluid [5], cholinesterase-positive (+) nerve fibers innervating the cornea are preferentially described in this section. Subsequently, human corneal subbasal nerve plexus demonstrated by IVCM is dealt with in the latest reports.

### 2.1. Specific Acetylcholinesterase- (AchE-) and Nonspecific Acetylcholinesterase- (NsAchE-) Positive Corneal Nerves

Since cholinesterase enzymes are present in sensitive end organs of the skin, the detection of corneal nerves with these enzymes is not surprising [6]. A histochemical demonstration by specific acetylcholinesterase method (AchE) (acetylcholine as substrate with inhibitors) or nonspecific acetylcholinesterase method (NsAchE) (butyrylthiocholine as substrate and BW284C51 as inhibitor of AchE-activity or acetylcholine as substrate without inhibitor) has been well used to visualize nerve fibers innervating the cornea in detail in many studies using several mammalian species: rabbit [6, 7], rat [8–13], and dog [11], including human [14]. The extracted corneas are examined in these studies on WMP [6, 7, 10–14] or flat (WMP in a broad sense [8, 9]) sectioned preparations for light microscopy [7, 8] and electron microscopy [7, 8]. A WMP is convenient for demonstration of the entire morphology and distribution pattern of corneal nerves.

#### 2.1.1. Cholinesterase-Positive Nerve Fibers in Animal Cornea

Both AchE (+) and NsAchE (+) nerve fibers were present in either stroma or epithelium [6–8]. AchE (+) nerve fibers in the subepithelial stroma are divided into fibrillary fibers (subepithelial nerve plexus) and perpendicularly penetrate into the epithelium at Bowman's membrane [6–8]. After penetration, subepithelial stromal nerve fibers with Schwann cells branch naked subbasal leash fibers [7, 8]. These leash fibers are running between epithelial cells (intraepithelial nerve) with beadings and finally form free nerve endings [7, 8].

NsAchE (+) nerve fibers appeared as the large nerve fiber bundles which make plexus anastomosing with each other in the stroma [12]. The NsAchE (+) stromal nerve fibers and their branches were quantitatively analyzed [9]. No significant differences in the stromal nerve fiber density were seen between each quadrant of the same cornea [9]. Stromal nerves are more closely innervated in the periphery than the central area of corneas [9, 12]. The density of stromal nerves (subepithelial nerve plexus) in the WMP was quantitatively estimated using computer morphometry and double staining methods (NsAchE and AuCl) [10]. The density of subepithelial nerves increased by 48% than only NsAchE or AuCl staining in the same cornea [10]. Visualization of epithelial nerves and their fine leash fibers were also enhanced by double staining. Age-related characteristics of NsAchE (+) corneal nerves (stromal nerve plexus) were studied in dogs [11]. NsAchE (+), thick 12–15 nerve bundles distributed evenly around limbus from which 2 nerve bundles of equal thickness branch run to the central area ramifying into thin nerve bundles. NsAchE (+) nerve fibers are also innervated in epithelial layer. Length of NsAchE (+) nerve fibers of adult dogs (1–7 years) was longer than those of young (until 1 year) and old dogs (more than 8 years) [11].

The nerve fiber density (mm/mm^2^) of NsAchE (+) stromal subepithelial plexus of rats with development (from 7 to 77 days after birth) was studied using WMP [12]. The density showed a decrease during development, indicating that the growth of NsAchE (+) nerve is not parallel with the enlargement of the cornea [12].

#### 2.1.2. Cholinesterase-Positive Nerve Fibers in Human Cornea

Entire normal architecture and distribution of human corneal nerves were studied with WMP by NsAchE method [14]. Over 44 thick stromal nerve trunks enter the cornea in a similar manner from the limbus and run towards the central portion [14]. Anterior stromal nerves peculiarly ramify at the mid-peripheral zone of the cornea. Passing through Bowman's membrane, they terminate as thick bulb from which many subbasal nerve (leash) fibers rise. The leash fibers vertically enter into the epithelium at the mid-peripheral cornea and run towards the corneal apex with a whorl-like or vortex structure [14]. The whirl-like pattern of subbasal nerves was also reported by immunostaining method using antibody for neuron specific *β*-tubulin in the mouse whole mount cornea [15, 16]. As a whole, the NsAchE (+) nerve fibers' architecture and distribution pattern in the human cornea are very similar to those of mammals, especially in mice [16].

The electron microscopic study revealed that the bundles of leash fibers consist of single or over 10 unmyelinated axons [17]. Leash fibers interconnect making subbasal plexus which is rich and complex in the central area than peripherally. From these leash fibers fine terminal fibers perpendicularly branch off and run between epithelial cells up to the ocular surface (intraepithelial nerve fibers). There are many free nerve endings (terminal boutons) en route to the surface.

### 2.2. Effects of Capsaicin on Corneal NsAchE-Positive and CGRP-ir Nerve Fibers

Some studies [18–20] reported that corneal nerve fibers stained with AuCl remarkably decreased following neonatal treatment with a sensory neurotoxin, capsaicin. The effects of neonatal pretreatment of capsaicin on the NsAchE (+) subepithelial stromal nerve fibers were investigated in adult rats [12, 13]. To our surprise, NsAchE (+) nerve fiber densities showed no significant differences compared with those of controls in any developmental ages [12]; these nerve fibers were insensitive to capsaicin. However, protein gene product 9.5 (PGP9.5: a marker protein of peripheral nerve fibers), immunoreactive (ir) intraepithelial nerve fibers or terminals decreased, causing a reduction of eye wiping by fourth-sixth to that of control by instillation of 100 *μ*M capsaicin onto the cornea [12]. According to the long survival times (4, 8, and 12 mos.) after subcutaneous (s.c.) neonatal capsaicin injection (50 mg/kg), various types of corneal lesions (neuroparalytic keratitis) occurred [13]. Occurrence of ulcers and irregular excess sprouting of fine NsAchE (+) nerve fibers were prominent in the animals 12 mos. after treatment. Even the short survival periods (5–75 days) after high dose of capsaicin application (total: 150 mg/kg), the pretreated animals showed corneal lesions and neovascularization, but not nerve fiber sprouting [13]. Thus, the longer survival times and the higher doses of capsaicin are key factors for inducing corneal lesion and neovascularization.

Neonatal intraperitoneal (i.p.) application of capsaicin induced the complete [20] (total dose: 150 mg/kg), or approximately 67% loss [21] (50 mg/kg, i.p.) of the CGRP-ir nerve fibers so that they seemed to have important roles in corneal sensory, blink, and trophic functions. Chronic keratitis and neovascularization severely occurred between 4 and 6 weeks after treatment with capsaicin [21].

### 2.3. Relations between Acetylcholine-Positive and Other Neurotransmitters-Positive Nerve Fibers in the Cornea

A similar pattern and density in the rat corneal stromal CGRP-ir nerves to those stained with NsAchE or AuCl suggested that majority of corneal stromal NsAchE (+) nerves are also CGRP-ir [22]. Similarly, AchE (+) thick nerve fiber bundles in the guinea pig corneal stroma coexisted with small CGRP-ir nerve fibers [23]; that is, thick nerve bundles are composed of AchE (+) and CGRP-ir nerve fibers. On the other hand, coexistence of neuropeptide galanin with SP [24] or SP with CGRP was demonstrated in the same corneal nerve fibers of mice [16] and pigs [24]. Therefore, capsaicin can deplete SP and CGRP from the corneal nerve fibers in the animals treated neonatally [4].

Another sensory neuropeptides neurokinin A (NKA, one of the tachykinin) and secretoneurin (generated by proteolysis of chromogranins) are present in the rat and human corneas [25, 26]. After neonatal capsaicin treatment, NKA- and secretoneurin-ir small neurons decreased by 50–60% in the rat TG [25, 26]. In addition to the classic neurotransmitters SP and CGRP, both NKA and secretoneurin may play a role in the corneal function.

Dense innervation of CGRP-ir and capsaicin receptor, transient receptor potential vanilloid 1- (TRPV1-) ir, subepithelial stromal nerve fibers was demonstrated in the rat cornea, though their colocalization with acetylcholine was not examined [27]. In addition, NsAchE-negative (−) but capsaicin-sensitive nerve fiber bundles making dense meshwork structures were seen in the rat cornea [28]. These meshwork structures are very similar to the leash fibers forming subbasal nerve plexus, showing a whirl-like pattern at the center of the cornea [28]. They were composed of both IB4-positive and TRPV1-ir fine nerve fibers [28]. Probably, SP (+), CGRP (+), TRPV1 (+), and IB4 (+) nerve fibers make meshwork bundles as subbasal leash fibers, but NsAchE (+) stromal nerve fibers never join in it. Thus, the presence of NsAchE (+) stromal nerve fibers and NsAchE (−) subbasal probable peptidergic leash fibers showing IB4 (+) or TRPV1 (+) indicate that these two groups are distinct populations from each other at least in the rat. The former is capsaicin-insensitive (resistant) and the latter capsaicin-sensitive.

Although there are many peptidergic, AchE (+), or nonpeptidergic IB4 (+) nerve fibers in small neurons in the DRG and TG of mammals, it should be noted that a considerable number of capsaicin-resistant small sensory neurons always remained within them after neonatal capsaicin treatment [25, 26, 29, 30].

**Figure 1:**
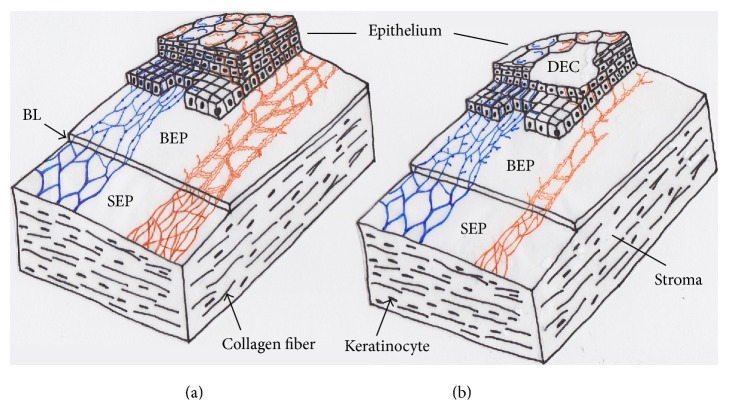
A putative architecture and distribution pattern of corneal nerve fibers of rats in normal (a) and their alterations after neonatal capsaicin treatment (b). Thick stromal subepithelial nerve fiber plexus are reactive to NsAchE. The NsAchE-positive (+) nerve plexus (indicated in blue) are resistant to capsaicin. On the other hand, NsAchE-negative (−) nerve fiber bundles showing dense interconnections (meshwork structure) reactive to neuropeptides and IB4 or TRPV1 seem to be subbasal leash fibers in rats cornea (indicated in orange). They are assumed to be destroyed (especially in the central portion) after neonatal capsaicin treatment (b), but NsAchE (+) nerves are never influenced by capsaicin. Probably, capsaicin-sensitive neurotrophic peptidergic nerve fibers are destroyed by neonatal capsaicin treatment resulting in the defect of epithelial cells (neuroparalytic keratitis) and desensitization to chemical or thermal stimuli. BEP: basal epithelial plexus of leash fibers, BL: Bowman's layer, DEC: defect of epithelial cells, and SEP: subepithelial (stromal) plexus.

The presumptive explanation on the morphology and distribution of corneal nerves and their changes after capsaicin treatment are illustrated in Figure 1 mainly based on the results of our studies [12, 13, 27, 28]. The report of Marfurt et al. [1] is also referred to because the distribution pattern of subbasal leash fibers is very similar to the meshwork bundles of subbasal leash seen in the rat cornea. Apparently, these subbasal leash nerve fibers sensitive to capsaicin have a crucial role for protection and repair the corneal tissue from chemical, mechanical, and infectious injuries. Numerous studies revealed that the loss of capsaicin-sensitive nerve fibers by capsaicin treatment induces corneal lesions in rodents [13, 18–21, 31–33]. Therefore, preservation and regeneration of capsaicin-sensitive nerve fibers in the cornea are a fundamental prescription for the maintenance of healthy corneal tissue.

### 2.4. Corneal Nerves by In Vivo Confocal Microscopy (IVCM)

From the beginning of 20th century, morphology of human corneal nerves has been progressively studied using IVCM [34–48]. A noninjurious way to examine the living human cornea is of special benefit in the use of IVCM [44]. However, an examination of the morphology of living corneal nerves is confined to the area of central cornea and subbasal nerve fibers [14]. Their innervation pattern and structure are very similar to the images in the previous reports described above. An appearance of whirl-like pattern in the central cornea of the subbasal nerve plexus is conspicuous [35, 40, 42] as well as demonstration with NsAchE [14] and immunostaining [15] methods. The IVCM has been used to clinically evaluate the morphological changes (i.e., tortuosity [45] loss or decrease [46, 47], and small fiber abnormalities [48]) of subbasal corneal nerves in various types of neuropathy.

## 3. Roles of TRP Channels in the Cornea

### 3.1. Overview of TRP Channels

There are two types of Ca^2+^ channels (i.e., voltage-dependent and receptor-operated channels). The transient receptor potential (TRP) channels are of the latter type. TRP channels can be classified into six main subfamilies: the TRPC (canonical), TRPV (vanilloid), TRPM (melastatin), TRPP (polycystin), TRPML (mucolipin), and TRPA (ankyrin) groups. TRP channels are expressed in almost every tissue and cell type and display an important role in the regulation of various cell functions [49].

The TRPC subfamily has seven members (TRPC1–7) [49–51]. It is activated by stimulation of G-protein-coupled receptors and receptor tyrosine kinase [49]. TPRC3, TRPC6, and TRPC7 are highly expressed in smooth and cardiac muscle cells [49]. The TRPV subfamily consists of TRPV1–6; TRPV1–4 have thermal sensitivity, and TRPV5 and TRPV6 uptake Ca^2+^ in the kidney and intestine [49]. The TRPM subfamily consists of TRPM1–8. Melastatin-1 is initially identified in melanoma cells so that the TRPM-1 is termed because of its homology with melastatin-1. TRPM channels are related to cell growth, differentiation, and programmed cell death [52]. TRPM2–4 and 8 have thermal sensitivity (threshold temperature 8–28°C) [53, 54]. The TRPP subfamily is isolated as causal factors of polycystic kidney disease. TRPP1 and TRPP2, which are associated with the interaction between cells and cell matrix, were found to coassemble at the plasma membrane and produce a new channel. The TRPML seems to participate in secretion of the stomach parietal cells, corneal epithelial cells, pancreatic acinar cells, hepatocytes, chondrocytes, and renal duct cells [55]. Mucolipidosis type IV (MLIV) is an autosomal recessive developmental disorder caused by mutations in the MLIV gene (MCOLN1), which encodes TRPML1 (mucolipin-1), a member of the TRPML subfamily [56]. The TRPA1 channel, also known as ANKTM1 or p120 protein [57], was found in a subset of sensory neurons coexpressing with TRPV1. TRPA1 channel can sense nociceptive cold at approximately 15°C [58]. Inflammatory agents mustard oil (allyl isothiocyanate: AITC, a constituent of horseradish and wasabi) and tetrahydrocannabinol (THC) excite over 20% of cultured rat TG neurons via ANKTM1, representing a subset (30–50%) of capsaicin-sensitive TRPA1-expressing small neurons having receptors for mustard oil and THC [59]. Bandell et al. [60] showed that both cinnamaldehyde (the main constituent of cinnamon oil used as flavor foods) and mustard oil activate only TRPA1.

The study on TRP channels in the ophthalmology area started from 2000, when TRPML1 was identified as a causal protein of mucolipidosis IV [55]. Since 2005, the relationship between retinal photosensory nerve cells and TRPC channel has attracted attention [61]. Moreover, Bellone et al. [62] reported that TRPM1 is correlated with congenital night blindness. Currently, the importance of TRP channels has been recognized in cornea, conjunctiva [63], ciliary body [64], and lens [65].

### 3.2. TRP Channels in Nociception of Cornea

For the first time, one of the ion channels TRPV1 is identified in small primary sensory neurons and later in nonneuronal cell types [24, 66–68]. It is cloned as the receptor for capsaicin (CAP) and activated by binding of various exogenous and endogenous compounds [50, 69–71]. When the channels of a subset of small sensory neurons are activated by CAP, they are opened and followed by inflow of the small molecule such as 5(6)-carboxyfluorescein- (FAM-) conjugated N-ethyl-lidocaine (QX-314) through the pore [72]. Structurally resembling vanilloids, such as resiniferatoxin (RTX) and olvanil, are also potent activators of this channel.

Ophthalmic branches of the trigeminal nerve are rich in distribution on the cornea (Figure 1) and conducive to touch or pain transmission from the tissue. Impairment of corneal sensitivity results in diminished blink and lacrimal reflexes. Our previous studies on corneal nerves suggested that the mesh-like fibers that were not stained with NsAchE are CAP-sensitive and make bunches of fine IB4- or TRPV1-positive nerve fibers, indicating that they are nociceptive in nature [24]. In agreement with this finding, numerous substances (e.g., components of shampoo and soap) causing eyes pain in daily life induce an influx of Ca^2+^ through TRPV1 in vitro [73, 74]. The number of eye-wipes in response to stimulation by CAP was significantly reduced in the RTX-treated group in rats [75]. Desensitization lasted a few days without affecting the healing process of the injured corneal epithelium as well as the blink reflex of the cornea [75]. Therefore, RTX is thought to be safe and useful drug in pain control after ophthalmic operation and eye disorders [75]. The results verified that TRPV1 plays an essential role in the corneal pain generation.

TRPV3 is transcribed from a gene adjacent to TRPV1, therefore, considered to form heteromeric channels with TRPV1. Naturally, TRPV1/3 heterotetrameric channels have different response properties from those of individual homotetrameric channels. Like TRPV1, TRPV3 expressed in keratinocytes and peripheral sensory neurons displays a marked sensitization (lowered threshold) to heat upon repeated stimulation [76]. Because significant delayed tail flick response (>50°C) and withdrawal latencies of hind paw at 55°C were seen in TRPV3-knockout (KO) mice, TRPV1 and TRPV3 are suggested to share a common noxious heat response [77].

TRPA1 channel is known to exist in the corneal nerve endings [24, 78–82]. There have been many reports on the TRPA1 concerning the pain; a pain in the trachea due to inflammation by smoking [83], inflammatory mediators such as prostaglandins (PG), and the intracellular alkalization [84, 85]. In addition to the pain, TRPA1 is associated with respiratory depression [86]. When the mice inhaled the materials, aerosolized hypochlorous acid or hydrogen peroxide (TRPA1 agonist), a respiratory frequency was remarkably decreased. The respiratory depressions were not seen in the TRPA1-deficient mice [86]. TRPA1 is activated by various factors, that is, an intracellular increase of Ca^2+^ concentrations, pH [84], cold [59], and mechanical force [87]. TRPA1 is also activated by chemicals such as allyl isothiocyanate (AITC; a component of wasabi) [59, 86], cinnamaldehyde (a component of cinnamon) [88], allicin (an ingredient of the garlic) [89], and formaldehyde [90]. Cinnamaldehyde causes burning and tingling sensations in humans following oral administration. Then Bandell et al. [60] suggested that the noxious and burning sensation by cold is transmitted through TRPA1 channel. Interestingly, TRPA1 has a sensitivity to isothiocyanates. Sensitivity to these electrophiles derives most likely from a reversible covalent bond [91]. The methyl isothiocyanate (MITC), sometimes called methyl mustard contained in a natural world, fends from animal predator [92]. MITC is used for agriculture to control nematodes, fungi, weeds, and insects. When MITC enters the atmosphere, it affects the human. Tear flow occurs after exposing the eye to MITC (tear gas effect) [92]. Thus, TRPA1 channel may be a sensor to protect the cornea from damage.

### 3.3. TRP Channels in Regeneration of Corneal Epithelial Cell

Renewal of the corneal epithelium is essential for corneal transparency and normal vision [93]. This layer has a barrier function against noxious stimuli [94]. The barrier properties are sustained by rigid joint structure between adjacent epithelial cells. The function of the corneal epithelium is maintained by replacing superficial terminally differentiated cell with proliferating basal cells undergoing migration into the upper layers of this tissue.

A growth factor (GF) is a naturally occurring substance capable of stimulating cell growth, proliferation and differentiation, and tissue healing. The GF includes the following types: epidermal growth factor (EGF), platelet-derived growth factor (PDGF), transforming growth factor (TGF), insulin-like growth factor (IGF), fibroblast growth factor (FGF), and nerve growth factor (NGF). The epithelial basal cell growth is controlled by EGF. An injury of the corneal epithelium stimulates the repair of the wound healing by EGF and other various different growth factors [95]. The EGF stimulates many signal pathways (namely, extracellular signal-regulated kinase: ERK, protein kinase A: PKA, protein kinase C: PKC, phosphoinositide 3-kinase: PI3-kinase, phospholipase C*ɤ*, phospholipase D, and inositol 1,4,5-trisphosphate: IP3) [96–98]. The role of these cascades is to increase intracellular calcium via the stimulation of capacitative calcium entry (CCE) [99]. The CCE means calcium influx due to activation by depletion in calcium stores. Therefore, it is also called store-operated calcium entry (SOCE).

Expression of TRPC4 is demonstrated in the corneal epithelial cell membrane by both immunocytochemistry and electron microscope studies [100]. TRPC4 is involved in the increment of cell proliferation by EGF [100]. EGF activates PLC-IP_3_ cascade. PLC-IP_3_ decrease the intracellular Ca^2+^ stores [79]. The decrease of Ca^2+^ stores works as feedback and provokes the increase of Ca^2+^ inflow into the cytoplasm by opening the store-operated Ca^2+^ channels (TRPC4) [99]. The increase of intracellular Ca^2+^ activates the downstream of the mitogen-activated protein kinase (MAPK) signal cascade and is followed by stimulation of PKA, PKC, Janus kinase/signal transducers and activators of transcription (JAK/STAT), and the phosphatidylinositol-3-kinase/protein kinase B (PI3-K/Akt). The activation of such MAPK signals can increase the cell proliferation and migration [98].

TRPV3 is broadly expressed in neuronal (DRG cells and central nervous system) and nonneural tissues including epidermis, keratinocyte, intestinal epithelial cells, and vascular endothelial cells [101]. The channel of TRPV3 is gated by warm temperatures at 32–39°C [74, 102, 103]. It is also activated by compress (heating pad) and ointment of the natural ingredients of camphor and the herbs (*Origanum vulgare*,* Syzygium aromaticum*, and* Thymus vulgaris* [104]). Aijima et al. [105] found that deficiency of TRPV3 decreased the reproduction of the epithelial cells compared with that of wild type. Recently, the functional expression of TRPV3 was demonstrated in human corneal epithelial cells (HCEC) [106]. Analysis of wound healing showed that the calcium ion inflow through TRPV3 in HCEC induces an increase of the epithelial cells proliferation [107]. TRPV3 seems to have an essential role in controlling the proliferation and the differentiation of epithelial cells via calcium entry.

Cannabinoids receptor subtype 1 (CB1) regulates many essential physiological processes including the control of neurotransmitter release, pain and analgesia, energy homeostasis modulation, and the immune cells function associated with guanosine triphosphate (GTP) binding protein [108, 109]. TRPV1 and CB1 are coexpressed and interact functionally in neuronal mesencephalic cultures [110], colon epithelium [111], myometrial smooth muscle cells [112], and primary sensory neurons [113]. The activity of TRPV1 in primary sensory neurons can be lowered by activation of CB1, though inhibitory effect of CB1 can be reduced in inflammatory conditions [113]. Yang et al. [114] showed the coexistence of TRPV1 and CB1 in HCEC. Injury of the corneal epithelium induces the release of endogenous activating substances, that is, anandamide and bradykinin, which are agonists of CB1 and TRPV1 activators, respectively [114]. When TRPV1 and CB1 are activated, proinflammatory cytokines (IL6 or IL8) are released from HCEC in the inflammatory condition. As a result, the activated TRPV1 and CB1 mediate cell proliferation and increase of migration through EGF receptor transactivation and MAPK/Akt-linked signaling pathway [115].

### 3.4. TRP Channels in Inflammation of Cornea

Nociceptive stimuli activate TRPV1 and induce proinflammatory cytokine release [115]. Zhang et al. [116] confirmed functional TRPV1 expression in the HCEC. The HCEC stimulated by CAP induced cationic electric current resulting in the increase of intracellular Ca^2+^ concentrations [117]. The release of proinflammatory cytokine (IL-6 and IL-8) is enhanced in HCEC by CAP [115, 117]. Such effects on HCEC are thought to be dependent on MAPK activation. Three inhibitors of MAPK pathways (U0126, SB203580, and SP600125) restrain the release of IL-6 and IL-8 [116]. Accordingly, TRPV1 channel contributes to the secretion of inflammatory mediators in the corneal epithelium [116].

EGFR-independent TRPV1-linked signal pathway mediates the release of IL-6 and IL-8 via TRPV1 stimulation [114]. On the contrary, the CB1 activation decreases the release of IL-8 induced by TRPV1 stimulation [114]. CB1 receptor is considered as an alternative medicine effective for the decrease of TRPV1-induced inflammation in corneal injury [114].

Expression of TRPV4 was demonstrated in peripheral sensory neurons, hypothalamus, and keratinocytes [128]. Recently, TRPV4 expression is also detected in HCEC [124]. There is a cross-talk between osmotic and heat stimuli; decline of osmotic pressure links to a decrease of temperature threshold [128]. TRPV4 channel is necessary for cell volume adjustment (regulatory volume decrease: RVD) after osmotic expansion. It is activated by exposure of TRPV4 agonist (4 alpha-phorbol-didecanoate: 4*α*-PDD) or hypotonic medium. An osmoregulation mechanism (RVD) is activated by an increase in intracellular calcium level through TRPV4. As a result, the volume of cells is kept constant [124]. Studies on TRPV4 expression using HCEC would provide a drug target therapy for preservation of corneal epithelial function during exposure to a hypotonic challenge or a thermal stress.

### 3.5. TRP Channels in Fibrosis or Scarring of Cornea

The healing process of corneal epithelium from alkali burn causes severe and permanent visual impairment with tissue inflammation, fibrosis, and scarring [118]. Loss of TRPV1 expression or the blockage of TRPV1 inhibited severe or persistent corneal inflammation and fibrosis/scarring [119]. Expression of transforming growth factor type 1 (TGF1) and other proinflammatory factors, that is, monocyte chemoattractant protein-1 (MCP-1) and IL-6, decreased in the alkali burned cornea of TRPV1-KO mice compared with those of the wild type [120]. MCP-1 and IL-6 are known to aggravate inflammation as chemoattractants of the inflammatory cells [119]. Inactivation of TRPV1 has a possibility for the potential medicine in improving the inflammatory/fibrogenic wound healing [119].

### 3.6. TRP Channels in Dry Eye

TRPM8 channel can be activated by cold temperature less than 25°C and by exposure to cooling compound such as menthol [53, 54]. The channels are represented in the cool-sensitive primary sensory neurons [120] and have specific electrophysiological properties [120, 129]. Madrid et al. [125] demonstrated TRPM8 channel expression in corneal nerve terminals. TRPM8 channel serves as “humidity detectors” sensing the slow temperature decrease due to evaporation of surface tears on eyes [126]. TRPM8-KO mice do not show a response to noxious cold stimulus but do show a reduction of basal tearing [125]. Moreover, the warming of cornea decreases the secretion of tears [126]. These findings indicate a contribution of TRPM8 for basal tearing in cornea.

Hirata et al. found the two classes of corneal nerves excited by drying the cornea: cold-sensitive (87%) and cold-insensitive (13%) neurons [127]. The application of the TRPM8 antagonist (N-(4-tertiarybutylphenyl)-4-(3-chloropyridin-2-yl)-tetrahydropyrazine-1(2H)-carbox-amide) on eyes induced a decrease of response to drying cornea by 45–80% [127]. Curiously, the treatment could never accomplish nonresponses. These results support an idea that the channel other than TRPM8 contributes to tear secretion as well. A candidate in place of TRPM8 channel for tear secretion is probably TRPV4 channel as mentioned above.

TRPV1 or an interplay between TRPV1 and TRPM8 is also relevant in dry eye diseases because TRPM8 activation is suppressed through activation of TRPV1 in HCEC [121]. Thyronamine as well as icilin is a cooling agent that directly activates TRPM8 at a room temperature. Moreover, thyronamine has an inverse effect on TRPM8 and TRPV1 activities when CAP is applied onto the cornea. While thyronamine activates TRPM8, TRPV1 stimulation by CAP is blocked, thereby protecting the dry eye [122].

Notably, TRPV1 can be activated by hypertonicity similar to patients with dry eyes. The activated TRPV1 induces proinflammatory cytokine (IL-6) release via MAPK signaling pathway [123] and leads to inflammatory condition [121], allowing of dry eye.

The role of each TRP channel is summarized in Table 1.

## 4. Pathogenesis and Treatment of Neurotrophic Keratopathy

### 4.1. Morphology of Corneal Nerves by In Vivo Corneal Confocal Microscopy (IVCM)

As described in the Introduction, the morphology of the human corneal nerves has been demonstrated in detail using in vivo corneal confocal microscopy (IVCM) examination [5, 27, 41]. IVCM is not yet an essential examination technique to determine treatment strategy. However, the relationship between morphological changes of corneal nerves elucidated by IVCM and corneal diseases is a field of study that will be developed in the future. Recently, IVCM is reported to be possible to detect the corneal nerve degeneration which related to the severity of neurological deficits in patients with mild multiple sclerosis [130]. The possibility of IVCM as an imaging biomarker for multiple sclerosis clinical course has also been reported [131]. In the future, the treatment outcome after corneal transplantation and/or the treatment result of ocular surface diseases such as dry eye may dramatically improve with the new treatment that takes into account innervation.

### 4.2. Neurotrophic Keratopathy

The corneal epithelial cells have trophic dependence on sensory nerves innervating the cornea (see Section 2.4). The extent of corneal lesions is in association with the destruction of peptide containing nerve fibers. Thus neuropeptidergic nerve fibers have neurotrophic activities to the corneal epithelial cells [132]. In other words, the corneal sensory nerves play an important role in homeostasis (maintenance) of the corneal epithelium. Neurotrophic keratopathy (NK) develops following the breakdown of the homeostasis. A diagnosis of NK depends on the past clinical history of the patient, because there is no specific corneal finding. Mackie classified NK into 3 clinical stages [133, 134]. We show pictures of NK in each stage after obtaining of informed consent from all patients for publication of this review.

**Figure 2:**
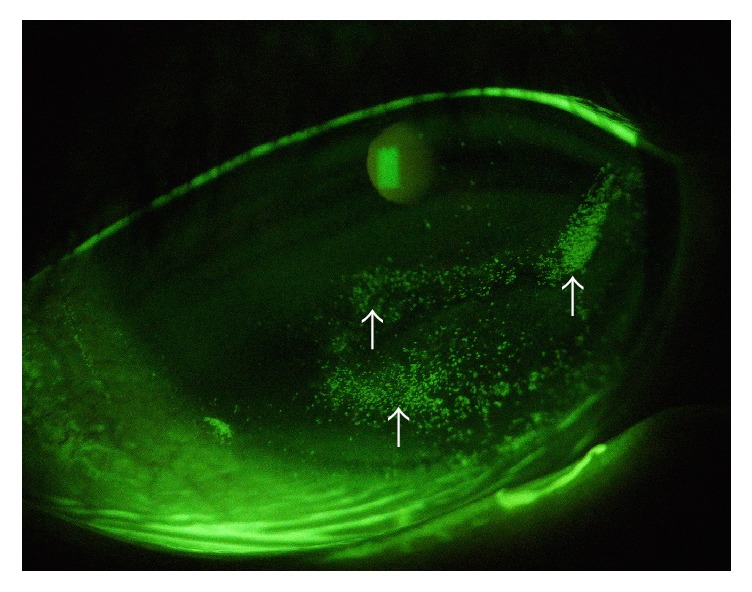
Stage 1 neurotrophic keratopathy (NK). Slit lamp microscope image stained by fluorescein. Superficial punctate keratitis (indicated by white arrows) occurred in a diabetic patient.

The first stage is characterized by fluorescein or Rose Bengal staining of the cornea. At this stage, superficial punctate keratitis (Figure 2), corneal epithelial hyperplasia, stromal scarring, and neovascularization are visible. The differential diagnosis of this stage includes many common ocular surface diseases, such as dry eye, contact lens wear, keratitis caused by blepharitis, and drug-induced keratopathy. These common diseases have certain symptoms, such as foreign body sensation, burning sensation, pain, and epiphora; however NK usually does not show any symptom.

**Figure 3:**
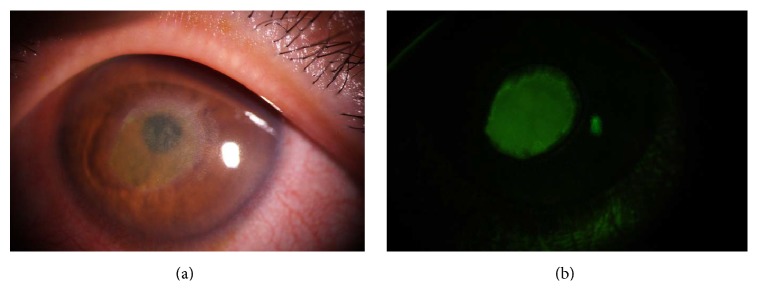
Stage 2 persistent epithelial defect (PED). Slit lamp microscope image (a). PED is located in the center of the cornea with cloudy stroma. Fluorescein staining image (b). The rolled edge of the PED can be seen.

The second stage shows a persistent epithelial defect (PED) (Figures 3(a) and 3(b)), that is, oval or circular in shape, and is often located in the superior half of the cornea. The edge of the PED is generally surrounded by loose epithelium that heals poorly and becomes rolled. Delayed PED healing causes a cloudy and oedematous cornea. However, the inflammation in the anterior chamber rarely occurs.

**Figure 4:**
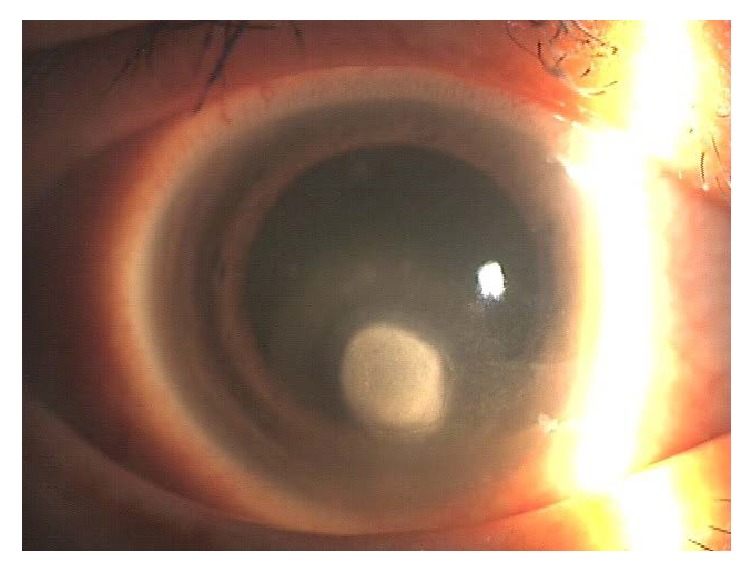
PED due to brain surgery and diabetes followed by infectious keratitis. The corneal epithelial disorder by NK led to methicillin-resistant* Staphylococcus aureus* keratitis (this picture is presented under the permission of Medical View Co., Ltd.).

The third stage shows a corneal ulcer with stromal thinning. In this stage, secondary infectious keratitis (Figure 4) and corneal perforation may occur. The second and the third stages can be confused with PED due to other pathophysiological causes or infectious keratitis. The most important key to distinguish stage 2 or 3 from PED caused by another disorder or infections keratitis is the inflammatory sign in the anterior chamber or a subjective complaint of severe eye pain.

### 4.3. Corneal Nerve in Diabetes Mellitus

In diabetes mellitus (DM), the incidence of corneal abnormalities is as high as that of retinal abnormalities. The density of nerve fibers in the diabetic patients has been reported to have a negative correlation with the duration of the diseases [135]. It is previously reported that DM patient has a decreased corneal sensitivity [136, 137] and a decrease in the nerve fiber bundles detected by IVCM which precedes corneal sensitivity impairment [137]. Reduction in neurotrophic stimuli induces a thin epithelial layer that may lead to recurrent corneal erosion [137]. Mocan et al. reported that patients with proliferative diabetic retinopathy had significantly lower subbasal nerve densities than patients with diabetes without retinopathy [138]. The latest report indicates that subbasal nerve changes precede other clinical and electrophysiological signs of neuropathy and corneal sensitivity testing as surrogate markers in the assessment of diabetic peripheral and cardiac autonomic neuropathy [139].

### 4.4. Diabetic Keratopathy

Diabetic keratopathy may be the most clinically problematic NK in ophthalmology. Neurotrophic corneal ulcers caused by DM have been known for long time [140]. It is thought that diabetic keratopathy is a representative of the corneal neuropathy due to diabetes [141]. DM patients have an increased risk of developing epithelial fragility, corneal recurrent epithelial erosion, abnormal wound healing, and infectious keratitis. The incidence of these corneal disorders has been estimated to be in 47–64% among DM patients [142].

### 4.5. Other Clinical Cases

#### 4.5.1. Refractive Surgery

During Laser in Situ Keratomileusis (LASIK) surgery, it is necessary to cut the corneal stroma to fashion the flap. Mechanical damage to the nerves results in LASIK-induced neurotrophic epitheliopathy (LINE), which has a serious role in dry eye following the surgery [143]. Severe destruction of subbasal corneal nerves due to flap formation is thought to cause post-LASIK dry eye [144]. In recent years, however, femtosecond laser assisted LASIK has been reported to have a lower incidence of LINE and decreased severity than the conventional microkeratome assisted LASIK [145]. In contrast to the fact that the subbasal nerve plexus appears to be fully regenerated at 5 to 8 months following photorefractive keratectomy (PRK) [146, 147], it remains incomplete for up to 5 years following LASIK [148]. Additionally, small incision lenticule extraction (SMILE) procedure using femtosecond laser to create an intrastromal lenticule is done for refractive surgery [144]. Since SMILE only makes small corneal incision (tunnel), there may be nonserious injury on corneal nerves. Then the SMILE is considered to prevent iatrogenic dry eye [144].

#### 4.5.2. Keratoplasty

With regard to penetrating keratoplasty (PKP), in which it is necessary to transect all corneal nerves, several articles [149, 150] report that the subbasal nerves are still tortuous and disorganize 40 years after surgery. High subbasal nerve densities in the corneal graft after PKP for keratoconus have also been reported [150]. The discrepancy between the previous observations may be due to differences in the causative disease, which affect the morphology and/or pathophysiology of the corneal nerve fibers.

#### 4.5.3. Postherpetic Neurotrophic Ulcer

Although the current treatment with antiviral medication reduces viral replication and also shortens both the disease course and the duration of symptoms, it helps maintain the herpes virus in latency [151]. Corneal sensitivity to mechanical stimulation decreases as the number of episodes of herpetic keratitis recurrence. NK with loss of corneal lustre and an irregularity of the corneal surface can develop in patients with recurrent herpetic epithelial keratitis because of impairment of corneal sensation [152, 153].

#### 4.5.4. Dry Eye

In Sjögren syndrome, most studies coincide with the fact that there is increased tortuosity of the subbasal nerves [154–156]. In non-Sjögren dry eye patients an increased tortuosity is also closely related to the severe dry eye [157].

### 4.6. Managements of NK

The therapeutic strategy to treat NK must consider the interactions between nerve regeneration and inflammatory pathways. Eye drops with preservatives should not be used. Benzalkonium chloride, widely used in many ophthalmic solutions, increases corneal inflammation and has a direct neurotoxic effect. Use of preservative free topical lubricants is essential for minimizing evaporation of the tears. Protecting the ocular surface with a soft contact lens bandage, tarsorrhaphy, and conjunctival flaps are effective for NK after stage 2. Eye drops containing peptides based on SP, a trigeminal nerve neurotransmitter, and insulin-like growth factor-1 (IGF-1), a multifunctional regulatory peptide that shares structural homology with proinsulin, have been reported to be effective in the prevention of superficial punctate keratitis in diabetic patients [158].

## 5. Conclusion

The morphology and distribution pattern of human corneal nerves examined by histochemical methods and in vivo confocal microscopy are very similar to those of animal model, especially to mice. Taken together, stromal nerve fibers are radially derived from thick limbal nerve fibers and then run towards the central area of the cornea forming subepithelial stromal nerve plexus. From the plexus several nerves branch off vertically passing through Bowman's membrane and run underneath the basal epithelial cells generally called subbasal leash fibers. The leash fibers make interconnections with each other (subbasal nerve plexus). The thin leash fibers perpendicularly give rise to intraepithelial nerve fibers and finally reach the upper epithelial cells leaving the beads of terminals. At the center of the cornea leash fibers show a whirl-like appearance. In the rat cornea, stromal subepithelial nerve fibers are nonspecific acetylcholinesterase- (NsAchE-) positive but thick meshwork bundles of subbasal leash fibers are never NsAchE-positive; that is, while the former are IB4- or TRPV1-negative and capsaicin-insensitive nerve fibers, the latter is NsAchE-negative but capsaicin-sensitive. These two nerve fiber groups seem to be separate populations at least in the rat cornea.

Until recently, a number of TRP channels (TRPV1-4, TRPA1, TRPC4, and TRPM8) are found in the corneal nerve fibers. Besides the thermal sensation, they have many functions such as protection from dry eye (humidity detectors sensing the evaporation of the corneal surface or inhibition from hypotonic challenge of the epithelium; TRPV4, TRPM8) and injury (TRPV1, TRPA1), wound healing (epithelial cell proliferation; TRPV1, TRPV3, and TRPC4), and contribution of pain and inflammation (TRPV1). The use of agonists or antagonists of these TRP channels are innovative strategy for the patients with various kinds of corneal diseases.

The causal relationships between morphological changes of subbasal corneal leash fibers and neurotrophic keratopathy (NK) are presented in the patients of diabetes mellitus, refractive surgery, keratoplasty, dry eye in Sjögren or non-Sjögren syndrome, and herpetic epithelial keratitis. The NK is classified into three stages, that is, appearances of superficial punctate keratitis, corneal epithelial hyperplasia, stromal scarring, and neovascularization (1st stage), a persistent epithelial defect (PED) (2nd stage), and a corneal ulcer with inflammatory sign and severe pain (3rd stage). Treatment with preservative free topical lubricants is crucial for management of NK. Eye drops of neurotransmitters, substance P or growth factors, are effective for prevention of superficial punctate keratopathy after surgery in diabetic patients.

## Figures and Tables

**Table 1:** Role of TRP channels in the cornea.

	TRPV1	TRPV3	TRPV4	TRPA1	TRPC4	TRPM8
Nociception	○			○		
Regeneration	○	○			○	
Inflammation	○		○			
Fibrosis or scarring	○					
Dry eye	○		○			○
Tissue location	Ner, Epi, End	Ner, Epi, End	Epi, End	Ner	Epi	Ner, Epi, End
Reference number	[24, 73–75, 109, 114–123]	[106, 107]	[124]	[24, 78–82]	[100]	[125–127]

○: TRP channels participating in physiological and pathophysiological conditions; Ner: corneal nerve, Epi: corneal epithelial cell, and End: corneal endothelial cell.

## Abbreviations

AchE: Acetylcholinesterase
AITC: Allyl isothiocyanate
BDNF: Brain-derived neurotrophic factor
CAP: Capsaicin
CB1: Cannabinoid receptor subtype 1
CCE: Capacitative calcium entry
CGRP: Calcitonin gene-related peptide
DM: Diabetes mellitus
DRG: Dorsal root ganglion
EGF: Epidermal growth factor
ERK: Extracellular signal-regulated kinase
FGF: Fibroblast growth factor
GTP: Guanosine triphosphate
HCEC: Human corneal epithelial cells
IB4: Isolectin B4
IGF: Insulin-like growth factor
IP3: Inositol 1,4,5-triphosphate
IVCM: In vivo confocal microscopy
JAK/STAK: Janus kinase/signal transducers and activators of transcription
KO: Knockout
LASIK: Laser in situ keratomileusis
LINE: LASIK-induced neurotrophic epitheliopathy
MAPK: Mitogen-activated protein kinase
MCP: Monocyte chemoattractant protein
MITC: Methyl isothiocyanate
MLIV: Mucolipidosis type IV
NGF: Nerve growth factor
NK: Neurotrophic keratopathy
NsAchE: Nonspecific acetylcholinesterase
NT-3: Neurotrophin-3
4*α*-PDD: 4 alpha-phorbol-didecanoate
PED: Persistent epithelial defect
PG: Prostaglandin
PGP 9.5: Protein gene product 9.5
PDGF: Platelet-derived growth factor
PI3: Phosphoinositide 3
PKA: Protein kinase A
PKC: Protein kinase C
PKP: Penetrating keratoplasty
PRK: Photorefractive keratectomy
QX-314: N-ethyl-lidocaine
RTX: Resiniferatoxin
RVD: Regulatory volume decrease
SEP: Subepithelial plexus
SMILE: Small incision lenticule extraction
SOCE: Store-operated calcium entry
SP: Substance P
TG: Trigeminal ganglion
TGF: Transforming growth factor
THC: Tetrahydrocannabinol
TRP: Transient receptor potential
TRPA: Transient receptor potential ankyrin
TRPC: Transient receptor potential canonical
TRPM: Transient receptor potential melastatin
TRPML: Transient receptor potential mucolipin
TRPP: Transient receptor potential polycystin
TRPV1: Transient receptor potential vanilloid 1
WMP: Whole mount preparation.
